# Case report: identification of one frameshift variant and two *in cis* non-canonical splice variants of *NEB* gene in prenatal arthrogryposis

**DOI:** 10.3389/fgene.2023.1220170

**Published:** 2023-08-31

**Authors:** Yuefang Liu, Juan Xu, Qiaoyi Lv, Zhe Liang, Lingling Li, Qiong Pan

**Affiliations:** ^1^ Genetic and Prenatal Diagnosis Center, Huai’an Maternity and Child Clinical College of Xuzhou Medical University, Huai’an, China; ^2^ Clinical Medical College, Yangzhou University, Yangzhou, China; ^3^ School of Medicine, Jiangsu University, Zhenjiang, China; ^4^ Family Planning Department, Huai’an Maternity and Child Clinical College of Xuzhou Medical University, Huai’an, China

**Keywords:** *NEB*, nemaline myopathies, case report, non-canonical splice variant, minigene

## Abstract

*NEB* mutation is associated with congenital nemaline myopathies. Here, we report a family with recurrent prenatal arthrogryposis. Trio whole exome sequencing (WES) disclosed three novel *NEB* (NM_001271208.2) variants including one paternal frameshift c.19049_19050delCA (p.Thr6350Argfs*14) and two double maternal variants in *cis* c. [24871G>T;24871-10C>G] (p. [Val8291Phe;?]). They are evaluated as “likely pathogenic (LP)”, “variant of uncertain of significance (VUS)”, and “VUS”, respectively. After further prediction, the c.24871G>T, c.24871-10C>G, and c.[24871G>T;24871-10C>G] were respectively genetically engineered into the three plasmids. Compared with their wild-type counterparts, the three plasmids all produced truncated transcripts, and also a significant proportion of the full-length transcripts, which allowed us to reclassify *NEB* c.24871G>T and c.24871-10C>G variants as LP. As far as we know, this is the first case carrying *NEB* allele-specific function of partial loss. This result helped the couple make informed reproductive choices and opt for assisted reproduction for future pregnancies. This study also increased awareness to the phenotype of prenatal nemaline myopathy and expanded the variant spectrum of *NEB*.

## 1 Introduction

Nebulin encoded by *NEB* is a structural component in the skeletal muscle sarcomere, which extends along the thin filament (Yuen and Ottenheijm, 2020). In addition to binding with the thin filament, a number of binding partners of nebulin have been reported such as *a*-actinin, *ß*-actinin, and titin (Chu et al., 2016; Yuen and Ottenheijm, 2020). Mutations in *NEB* might alter the binding affinity of these binding partners and associate with autosomal recessive nemaline myopathy (NM). NM is a heterogeneous group of congenital myopathies characterized by skeletal muscle weakness and the presence of electron dense protein accumulations (nemaline rods) on muscle biopsy (Sewry et al., 2019). Histopathological features have a major role in directing NM diagnosis. However, nemaline rods may be absent in milder, distal NM (Wallgren-Pettersson et al., 2007) and rarely nemaline rods may also occur in aging muscles and muscle cells suffering from metabolic stress (Cassandrini et al., 2017). Therefore, next-generation sequencing is of utmost importance for the diagnosis of NM. At present, dominantly or recessively inherited mutations in at least 13 genes are responsible for NM, which include nebulin (*NEB*), alfa-actin (*ACTA1*), alfa-tropomyosin (*TPM3*), beta-tropomyosin (*TPM2*), troponin T1 (*TNNT1*), and cofilin 2 (*CFL2*) (Moreau-Le Lan et al., 2018). *NEB* is the most common mutated gene causing NM, accounting for approximately 50% of the reported cases (Ottenheijm et al., 2010). The most common types of variants in *NEB* are splice-site mutations (34%) (Lehtokari et al., 2014).

Precise pre-mRNA splicing depends on the presence of consensus *cis* sequences. The *cis* sequences include donor (5′) and acceptor (3′) splice sites (±1/±2), branch point (YUNA^*^Y, −9 and −400 downstream from the acceptor site), polypyrimidine tract sequences (PyT) (Y_n_, −5 and −40 bp downstream from the acceptor site), exonic and intronic splicing enhancers (ESE and ISE), and exonic and intronic splicing silencers (ESS and ISS) (Anna and Monika, 2018). The most classical ±1/±2 splicing mutations are often classified as pathogenic. However, the exact localization of branch point, PyT, splicing silencers, and enhancers are difficult to determine. Therefore, mutations disrupting branch point, PyT, splicing silencers, and enhancers are hard to identify and often classified as variants of uncertain significance (VUS).

Bioinformatics algorithms have been developed to assess the possible splicing effect of the identified variants. However, it should be highlighted that functional studies must be performed to verify the exact effect of the specific mutation. Sanger sequencing of RNA/cDNA from the patient’s sample is the simplest and most effective method to assess splicing abnormalities. However, fresh RNA sample from affected individuals is rarely available and potential splicing mutation can be easily missed out if nonsense-mediated decay (NMD) is present. The minigene system has been proved to be an effective tool for evaluating the splicing variants *in vitro* (Anna and Monika, 2018; Zhang et al., 2021). In the minigene assay, the amplified fragment containing the interested exon, flanked by upstream and downstream intronic sequences with and without mutations, is cloned into a special expression plasmid enabling the analysis of the pre-mRNA splicing.

In the current article, we describe a family with recurrent prenatal congenital arthrogryposis in the first and third pregnancies. Trio-WES identified three novel *NEB* variants that included one paternal variant in exon 122 (NM_001271208.2:c.19049_19050delCA (p.Thr6350Argfs*14)) and double maternal variants NM_001271208.2:c. [24871G>T; 24871-10C>G] *in cis* in exon 178 and intron 177, respectively. The study of splicing impact of c.24871G>T and c.24871-10C>G variants was performed by both *in silico* analysis and *in vitro* minigene assay. Finally, the c.24871G>T and c.24871-10C>G variants were reclassified as likely pathogenic (LP).

## 2 Materials and methods

### 2.1 Patients

It was a non-consanguineous marriage for the family from China. The family history revealed that prenatal arthrogryposis occurred in the mother’s first and third pregnancies, while the second resulted in a healthy girl. For the first pregnancy, a genetic etiology was not evaluated and no available DNA was preserved.

The peripheral blood sample from the couple and tissue sample from the aborted fetus were collected for trio-WES after having received the expressed consent from both the guardians. The research protocol was approved by the local ethics committee of Jiangsu Huai’an Maternity and Child Healthcare Hospital (2021042).

### 2.2 Genetic analysis

The whole exomes and intron regions, 20 bp preceding the exons, were captured by using the Roche KAPA HyperExome. Whole exome sequencing (WES) was conducted by the MGISEQ-2000. The raw data for the fetus, father, and mother was 24288.26, 23110.65, and 18999.09 Mb, respectively. The sequencing depth of the target area for each sample was 341 X, 321 X, and 256 X. The proportion of an average depth >20 X in the target area was all about 99%. The obtained sequences were aligned to the human genome GRCh37/hg19 reference sequence by using the BWA (Burrows–Wheeler Aligner) software. A BAM (binary sequence alignment map format) file was produced via the Picard software. The GATK4 (Genome Analysis Toolkit) RealignerTargetCreator software and HaplotypeCaller software were used to adjust the sequence, extract variants, and generate VCF (Variant Call Format) files. The ANNOVAR software was used to filter and annotate the variant. The deleteriousness of the variants was assessed according to the American College of Medical Genetics (ACMG) 2020 standards and guidelines.

### 2.3 *In silico* prediction of splice-affecting nucleotide variants

RNA Splicer (https://rddc.tsinghua-gd.org/search-middle?to=SplitToolModel), varSEAK (https://varseak.bio/index.php), SpliceAI (https://spliceailookup.broadinstitute.org/), and Human Splicing Finder (HSF) (https://www.genomnis.com/access-hsf) were used to evaluate the splice effect of the nucleotide variants. The AI model of the RNA Splicer was implemented by the machine learning method combined with the permutation test. It predicts the alternative splicing site of the mRNA sequence by learning the canonical splicing pattern. SpliceAI is based on artificial neural networks and accurately predicts splice junctions from an arbitrary pre-mRNA transcript sequence, enabling precise prediction of non-coding genetic variants that cause cryptic splicing (Jaganathan et al., 2019). The HSF system is dedicated to the identification of all splicing signals that include acceptor and donor splice sites, branch points, and auxiliary splicing signals (Desmet et al., 2009).

### 2.4 Minigene splicing assay

Two pairs of nested primers 239049-NEB-F and 241692-NEB-R, and 239416-NEB-F and 241369-NEB-R were designed for nested PCR by using the gDNA of the mother as the template. Four minigenes that included wild type (wt), *NEB* c.24871G>T (mut 1) in exon 178, *NEB* c.24871-10C>G (mut 2) in intron 177, and *NEB* c.[24871G>T;24871-10C>G] (mut 3) were constructed as follows.

#### 2.4.1 Strategy of pcMINI-N-wt/mut 1/mut 2/mut 3 plasmids

**FIGURE 1:**
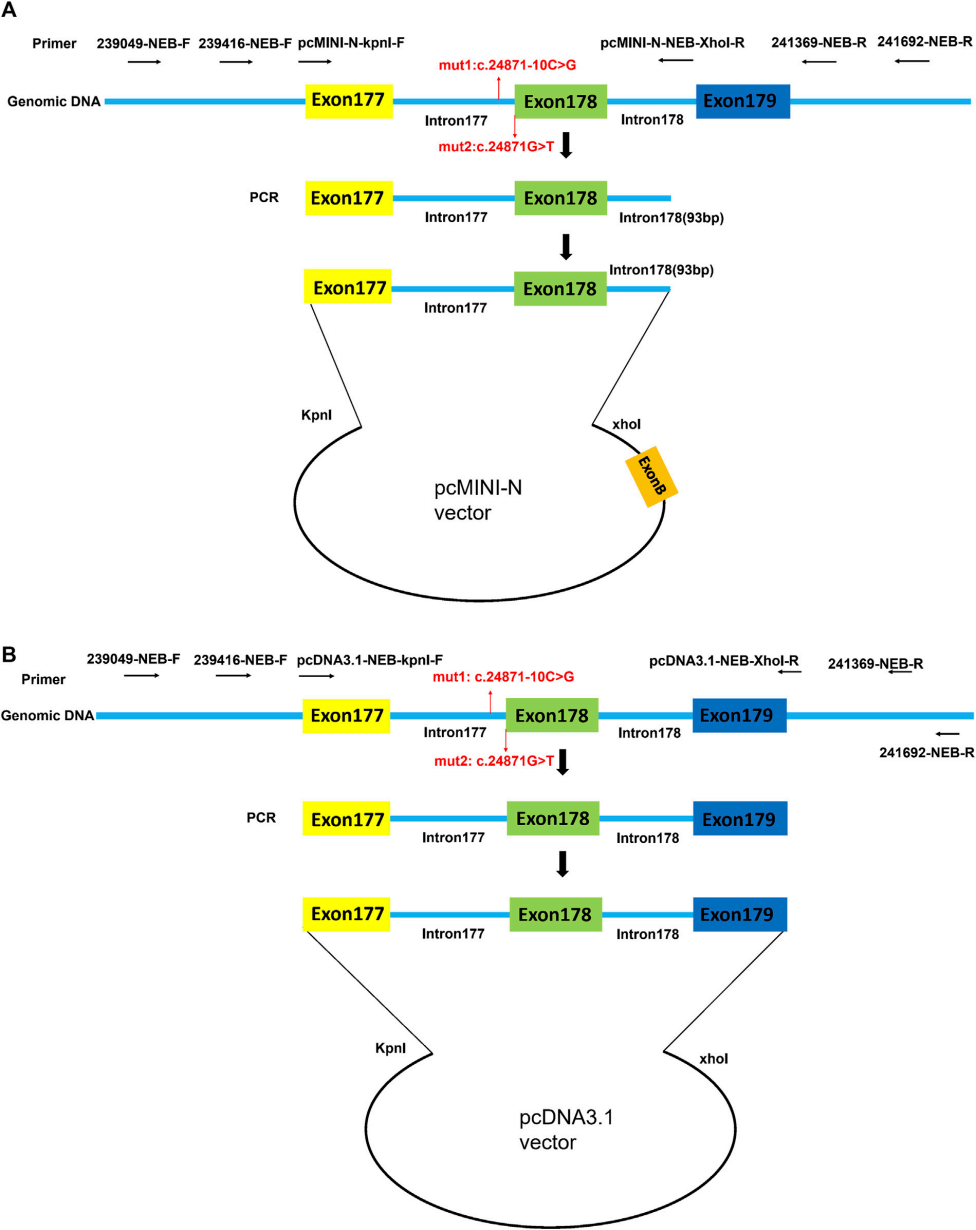
Schematic diagram of pcMINI-N vector and pcDNA3.1 vector construction for minigene assay. **(A)** Target gene region covering exon177 (93bp)-intron177 (858bp)-exon178 (108bp)-partial intron178 (93bp) of *NEB* was inserted into the pcMINI-N vector. **(B)** Target gene region covering exon177(93bp)-intron177(858bp)-exon178(108bp)-intron178(105bp)-exon179(184bp) of *NEB* was inserted into the pcDNA3.1 vector. Exon B is a universal exon on the pcMINI-N vector.

By using the nested PCR product as the template, the target gene region covering exon177(93bp)-intron177(858bp)-exon178(108bp)-partial intron178(93bp) of the *NEB* was amplified by primers pcMINI-N-NEB-KpnI-F and pcMINI-N-NEB-XhoI-R. The obtained products were inserted into the pcMINI-N vector (Figure 1A) and the monoclonal expression of pcMINI-N-wt and pcMINI-N-mut 3 was selected. In order to obtain mut 1 minigene, wt minigene was first amplified by two pairs of primers (pcMINI-N-NEB-KpnI-F and NEB-mut 1-R, and NEB-mut 1-F and pcMINI-N-NEB-XhoI-R) separately. Then, mixed products were amplified by pcMINI-N-NEB-KpnI-F and pcMINI-N-NEB-XhoI-R. The mut 2 minigene was constructed by using the same experimental method as previously mentioned.

#### 2.4.2 Strategy of pcDNA3.1-wt/mut 1/mut 2/mut 3 plasmids

By using the nested PCR product as the template, the target gene region covering exon177(93bp)-intron177(858bp)-exon178(108bp)-intron178(105bp)-exon179(184bp) of *NEB* was amplified by primers pcDNA3.1-NEB-KpnI-F and pcDNA3.1-NEB-XhoI-R. The obtained products were inserted into the pcDNA3.1 vector (Figure 1B) and the monoclonal expression of pcDNA3.1-wt and pcDNA3.1-mut 3 was selected. In order to obtain mut 1 minigene, wt minigene was first amplified by two pairs of primers (pcDNA3.1-NEB-KpnI-F and NEB-mut 1-R, and NEB-mut 1-F and pcDNA3.1-NEB-XhoI-R) separately. Then, the mixed products were amplified by pcDNA3.1-NEB-KpnI-F and pcDNA3.1-NEB-XhoI-R. The mut 2 minigene was constructed by using the same experimental method as previously mentioned.

Subsequently, the plasmids were validated through Sanger sequencing. Wild and mutant vectors were transfected into the HEK293 cell separately. With total RNA extracted and reverse-transcribed into cDNA after 48 h of transfection, reverse transcription polymerase chain reaction (RT-PCR) was triggered by a primer pair of pcMINI-N-F/pcMINI-N-R or pcDNA3.1-F/pcDNA3.1-R. Finally, the cDNA products were examined using 1% agarose gel electrophoresis and further confirmed by Sanger sequencing. To substantiate the conclusion drawn in this study, the assay was repeated in another HeLa cell line by using the same experimental method as previously mentioned. The sequence of all primers is listed in Supplementary Table S1.

## 3 Results

### 3.1 Clinical features and genetic findings

The birth from the first pregnancy was not until 1 week after the due date. The amniotic fluid was found to be cloudy. The fetus died shortly after birth from respiratory insufficiency. On gross examination, the newborn was complicated by restricted hip movement, hypotonia, weakness, thin and flexed finger, and club foot. A genetic etiology was not evaluated, and no available DNA was preserved.

**FIGURE 2:**
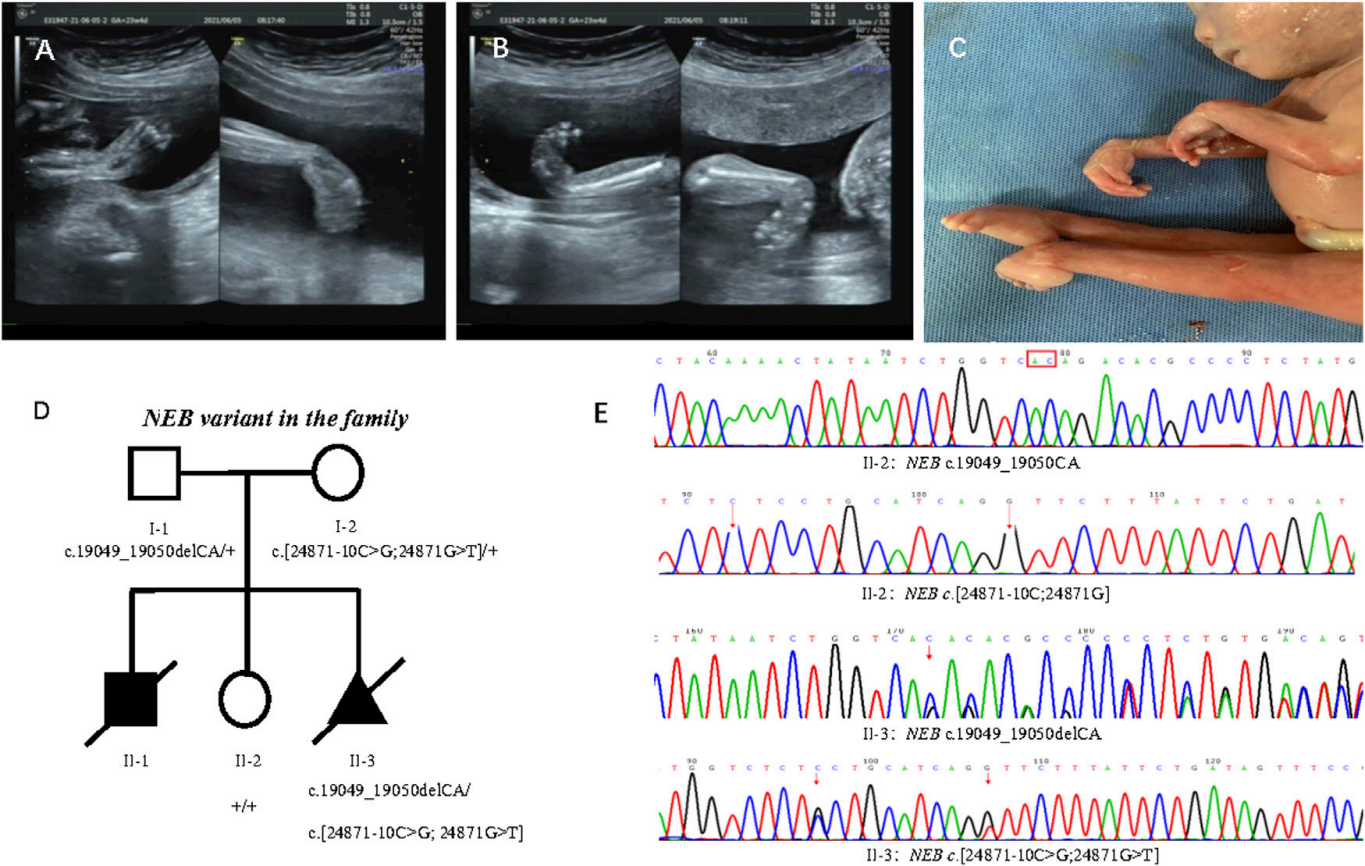
Phenotype of the proband and family segregation analysis. **(A)** Wrist flexion contracture by prenatal ultrasounds. **(B)** Severe equinovarus deformities of the feet by prenatal ultrasounds. **(C)** Extension of both legs, and congenital contractures affecting wrists and ankles. **(D)** Family segregation analysis of *NEB* variant. **(E)** *NEB* variant in healthy sister (II:2) and affected fetus (II:3) by Sanger sequencing.

In 2021, the third pregnancy was conceived spontaneously. Due to the previous adverse pregnancy, the family was referred to first trimester screening at 13 weeks’ gestation. Ultrasound revealed normal nuchal translucency. In the 22nd week, ultrasound findings were consistent with the previously affected fetus that included wrist flexion contracture, severe equinovarus deformities of the feet, and extension of both legs (Figures 2A, B). Amniocentesis was done after genetic counseling, and the Affymetrix CytoScan 750K SNP array used for chromosome analysis showed no abnormality. The pregnancy was terminated at 23 weeks of gestation because of suspected inherited disease. *Postmortem* examination revealed congenital contractures affecting hands, wrists, feet, and ankles (Figure 2C). After genetic counseling, trio-WES was chosen for further detection.

Trio-WES disclosed compound heterozygous variants of *NEB* (NM_001271208.2) that included one paternal variant in exon 122: chr2:152417774_152417773delCA; c.19049_19050delCA (p.Thr6350Argfs*14) and double maternal variants *in cis* in exon 178 and intron 177: chr2:[152349008G>T;152349018C>G];c.[24871G>T; 24871-10C>G] in the reference genome GRCh37/hg19, which were confirmed by Sanger sequencing (Figures 2D, E). The transcript produced by paternal *NEB* c.19049_19050delCA variant was predicted to undergo NMD or be a truncated protein resulting in a loss of function (LOF) (PVS1). So far, this variant is not present in the ExAC, 1000 Genomes Project, and gnomAD (PM2-Supporting), and it is classified as LP according to the ACMG guidelines (PVS1+ PM2-Supporting). The *NEB* c.24871G>T (p.Val8291Phe) and c.24871-10C>G variants from the mother were not present in the ExAC, 1000 Genomes Project, and gnomAD (PM2-Supporting) and has been classified as VUS according to the ACMG guidelines (PM2-Supporting+PM3).

The family segregation analysis showed that neither maternal c.[24871G>T;24871-10C>G] nor paternal c.19049_19050delCA were detected in the healthy daughter by Sanger sequencing (Figure 2E).

### 3.2 *In silico* prediction of effect of *NEB* c.24871G>T and c.24871-10C>G variants

**FIGURE 3:**
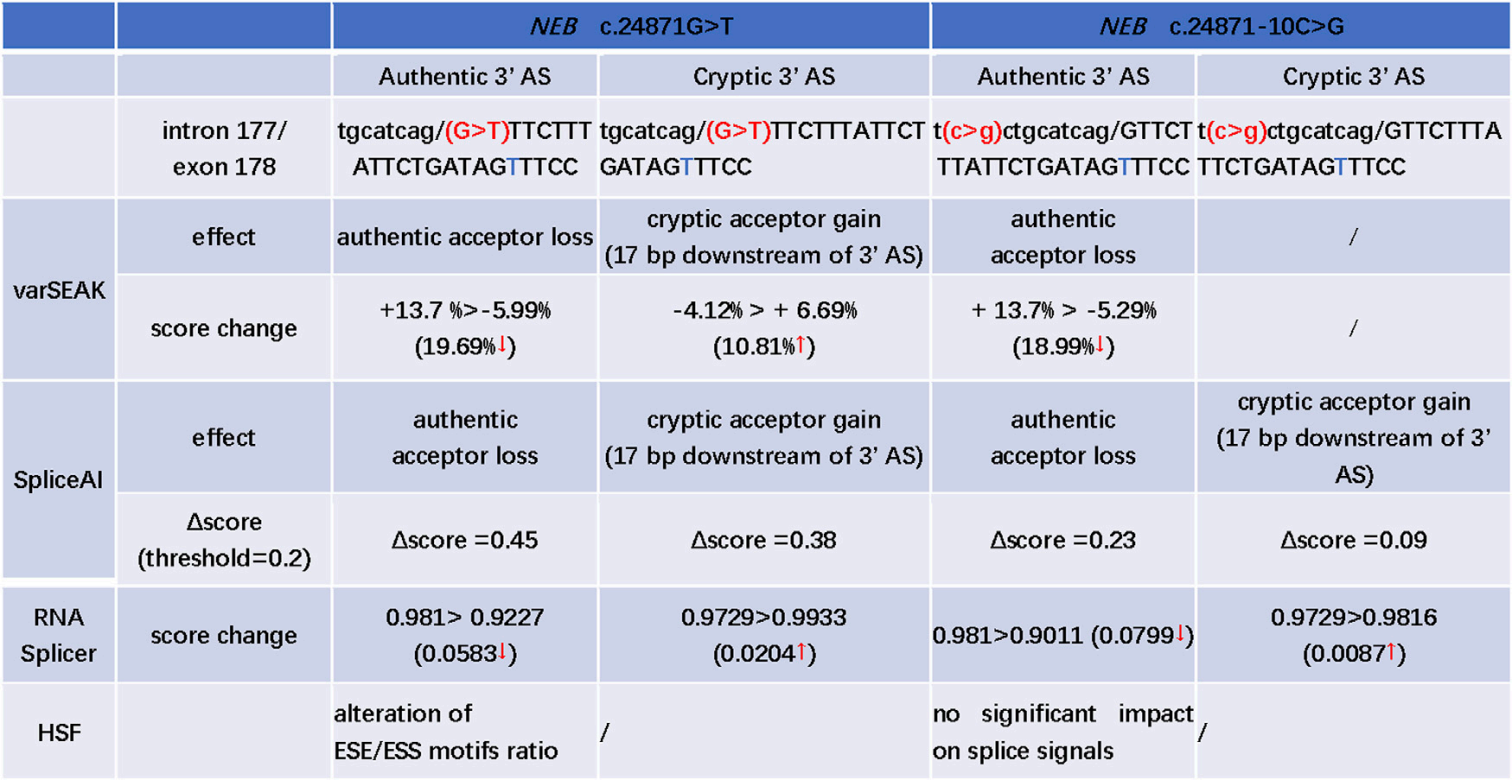
*In silico* prediction of possible splicing effect of the *NEB* c.24871G>T and c.24871-10C>G variants. AS: acceptor site; “t” marked with blue: a cryptic acceptor site (17 bp downstream of 3′ splice site); “>” is used to describe the score change between wild type and variant; ↑: the predicted score increased after mutation; ↓: the predicted score decreased after mutation; the Δscore in SpliceAI, ranging from 0 to 1, indicates the probability that the mutation affects normal splicing and the recommended threshold is >0.5; 0.2 < Δscore < 0.5: may affect splicing; >0.8: being very likely to affect splicing.

The c.24871G>T was the first base of exon 178. SpliceAI, varSEAK, RNA Splicer, and HSF programs were used to evaluate the splice effect of *NEB* c.24871G>T and c.24871-10C>G mutations. For c.24871G>T variant, SpliceAI, varSEAK, and RNA Splicer all predicted a loss of authentic acceptor site and activation of cryptic acceptor site at 17 bases downstream of the start of exon 178 (Figure 3). The HSF analysis showed that c.24871G>T altered the ESE/ESS motifs' ratio (Figure 3). For c.24871-10C>G, SpliceAI, varSEAK, and RNA Splicer all predicted a loss of authentic acceptor site. The lower confidence of cryptic acceptor gain at 17 bases downstream of the start of exon 178 was indicated by SpliceAI and RNA Splicer (Figure 3).

### 3.3 *In vitro* functional analysis

**FIGURE 4:**
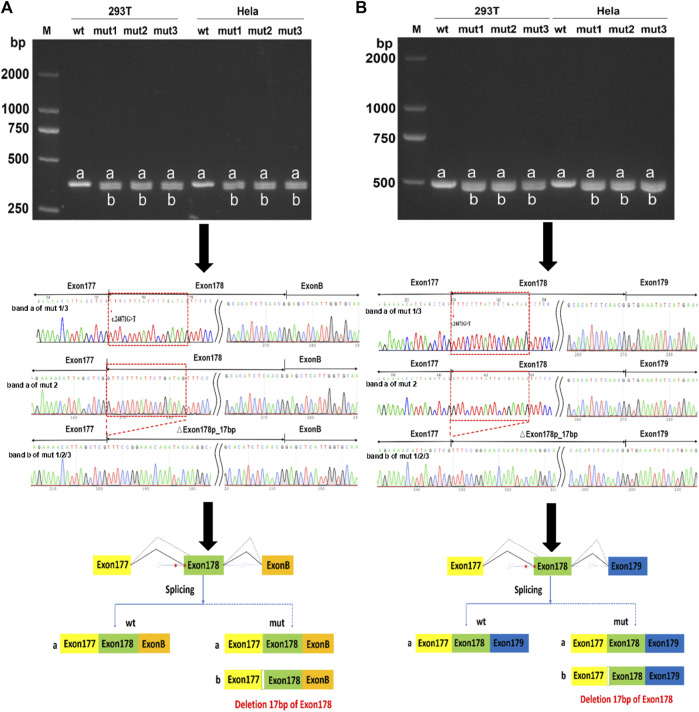
*In vitro* minigene assay of wild type (wt), *NEB* c.24871G>T (mut 1), *NEB* c.24871-10C>G (mut 2), and *NEB* c.[24871G>T; c.24871-10C>G] (mut 3) variants. **(A)** Gel electrophoresis and Sanger sequencing confirm that mut 1, 2, and 3 all produced a full-length transcript (band a) and a shorter transcript (band b) with 17 bp deletion downstream of the start of exon 178 in pcMINI-N vector. **(B)** Gel electrophoresis and Sanger sequencing confirm that mut 1, 2, and 3 all produced a full-length transcript (band a) and a shorter transcript (band b) with 17 bp deletion downstream of the start of exon 178 in pcDNA 3.1 vector.

An *in vitro* minigene splicing assay was performed as both c.24871G>T and c.24871-10C>G were predicted to disrupt splicing. When compared with the wt minigene, *NEB* c.24871G>T (mut 1), *NEB* c.24871-10C>G (mut 2), and *NEB* [c.24871G>T; c.24871-10C>G] (mut 3) all produced not only truncated transcripts but also a significant proportion of full-length transcripts (Figures 4A, B). As revealed by the Sanger sequencing, the loss of the first 17 bases of exon 178 was detected in truncated transcripts (NM_001271208.2:r.25063_25079del) (Figures 4A, B). The newly formed transcript was predicted to produce a prematurely truncated protein p.(Val8291Phefs*35). The *NEB* c.24871G>T and c.24871-10C>G variants were reclassified as LP according to the ACMG guidelines (PM2-Supporting+PM3+PS4).

## 4 Discussion

*NEB* is an extremely large gene with 183 exons (Donner et al., 2004) and about 7% of all coding variants are pathogenic variants, which indicates that nebulin tolerates substantial changes in its amino acid sequence (Lehtokari et al., 2014). Exon 55 deletion is relatively common among Ashkenazi Jewish (Lehtokari et al., 2009; Lehtokari et al., 2014). Other mutations are distributed throughout the gene without any mutation hotspot. The most common types of variants are splice-site mutations (34%), followed by frameshift mutations (32%), nonsense mutations (23%), and missense mutations (7%) (Lehtokari et al., 2014). The c.19048_19057del (p.Thr6350fs) variant in exon 122 has been reported as the Finnish founder mutation (Lehtokari et al., 2014). In our study, the proband shared the similar frameshift c.19049_19050delCA (p.Thr6350Argfs*14) variant in *NEB* exon 122 altering the amino acid sequences at the same location.

Splicing mutations can be briefly divided into five categories based on the variant location and splicing alterations (Wimmer et al., 2007; Anna and Monika, 2018). Mutation in the canonical acceptor and donor sites (±1/±2) leads to exon skipping (type I) or activation of cryptic splice sites leading to the inclusion of an intron fragment or exon fragment skipping (type IV). Deep intronic mutation creates a new splice site resulting in the pseudo exon inclusion (type II). Mutation within the exon creates a new splice site causing the loss of an exon fragment (type III) or disrupts ESE causing exon skipping (type V). The exon mutation causing splicing alterations can be easily misclassified as the synonymous, missense, or nonsense variant. In our study, the c.24871G>T variant in the *NEB* gene was misclassified as a missense variant (p.Val8291Phe). The HSF predicted that the c.24871G>T variant changed the ESE/ESS motifs' ratio. VarSEAK, SpliceAI, and RNA Splicer all indicated authentic acceptor site loss and gain of a cryptic acceptor site located in 17 bp downstream of the start of exon 178. Consistent with the *in silico* prediction, the minigene showed that the c.24871G>T variant resulted in two mRNA isoforms: one properly spliced and containing the substitution that could lead to missense change at the protein level (p.Val8291Phe), and the other that loses the first 17 bases of exon 178 that is classified as type III mutation.

As mentioned in the Introduction section, the splicing process is dependent on the presence of the branch site and PyT sequences. The PyT sequences are enriched in pyrimidine nucleotides and usually located −5 and −40 bp downstream from the acceptor site (Vaz-Drago et al., 2017; Anna and Monika, 2018). It has been recommended that the functional analysis should be performed to confirm the splicing effect of an intronic variant near the acceptor splice site (Lewandowska, 2013). In our study, the *NEB* c.24871-10C>G variant was 10 bp upstream from the acceptor site. *In silico* analysis of possible splicing effect of the c.24871-10C>G variant drew conflicting results. Furthermore, the minigene assay confirmed that the c.24871-10C>G variant activated an alternative 3′ acceptor site at 17 bases downstream of the start of exon 178 competing with authentic splice acceptor sites, indicating that at least one allele produces normal nebulin. Our results highlight that functional studies are necessary. However, the minigene assay was limited because the effect of a specific splicing mutation also depends on the tissue type in which the primary transcript is expressed at different levels (Anna and Monika, 2018). In summary, this is the first case with *NEB* allele-specific function of partial loss, and a definite diagnosis of NM in this case was achieved. We regard it likely that paternal *NEB* c.19049_19050delCA mutation and maternal *NEB* c. [24871G>T; c.24871-10C>G] variants may be the causative mutation of the first affected pregnancy.

The *NEB* gene comprises 183 exons and exons 63–66, 82–105, 143–144, and 166–177 are alternatively spliced (Donner et al., 2004). The exons 166–177 give rise to at least 20 different transcripts in the adult human tibialis anterior muscle alone. Exons 178–183 are present in all these 20 different transcripts. The *NEB* gene consists of 185 multiple repeat modules that contain the actin-binding domain. The C-terminal was anchored in the Z-disk region of the sarcomere and contains multiple repeat module 182–185 and a conserved serine-rich homology domain (SH3) (Yuen and Ottenheijm, 2020). The SH3 domain located at the C-terminal binds a large number of muscle proteins such as a-actinin myopalladin, titin, and desmin (Chu et al., 2016; Yuen and Ottenheijm, 2020). In our study, *NEB* c.24871G>T and c.24871-10C>G introduced a novel cryptic acceptor splice site in exon 178 and premature termination codons in exon 179, which might lead to the loss of the C-terminal in truncated transcripts and further affect its ability to bind muscle proteins.

Severe congenital form of nemaline myopathy onset at or before birth and the inclusion criteria are no spontaneous movements, no spontaneous respiration, or with arthrogryposis multiplex congenita (AMC) (Yonath et al., 2012; Bohm et al., 2013). AMC refers to the development of multiple joint contractures affecting two or more areas of the body prior to birth (Skaria et al., 2019). Gestational diabetes mellitus, maternal infections, fetal crowding, and abnormal neuromuscular disorders are fairly a common cause of AMC (Skaria et al., 2019). A genetic cause can be identified in about 30% of affected fetuses. Prenatal imaging is crucial in early diagnosis by identifying fetal movement limitations and the presence of club foot. In a review of 10 fetuses with NM, polyhydramnios and decreased fetal movement were commonly detected, ranging from week 13 to the third trimester. Club feet were often observed around week 20 (Yonath et al., 2012; Bohm et al., 2013; Rocha et al., 2021). After birth, arthrogryposis, club foot, undescended testes, respiratory failure, and muscle weakness were frequently observed. In conclusion, when ultrasonographic features such as polyhydramnios, decreased fetal movements, club feet, and arthrogryposis are noted during pregnancy, a congenital myopathy should be considered in the differential diagnosis. In our research, prenatal detectable onset of symptom was club feet at around week 20.

In summary, we have reported a prenatal arthrogryposis case in mid-term pregnancy caused by compound heterozygotes in *NEB* that included two splice variants *in cis*. The minigenes assay reclassified them into LP variants. Once again, the minigenes assay has been proven to be a useful tool to provide key data for the interpretation of potential spliceogenic variants.

## Data Availability

The data presented in the study are deposited in the China National Gene Bank Data Base (CNGBdb) repository, accession number CNP0004274.
